# A Touch on Musical Innovation: Exploring Wearables and Their Impact on New Interfaces for Musical Expression

**DOI:** 10.3390/s24010250

**Published:** 2023-12-31

**Authors:** David Wexler, Joanne Yip, Ka-Po Lee, Xiaolu Li, Yiu-Hong Wong

**Affiliations:** 1 School of Fashion and Textiles, The Hong Kong Polytechnic University, Hung Hom, Kowloon, Hong Kong SAR, China; d.wexler03@gmail.com (D.W.); maple.lee@connect.polyu.hk (K.-P.L.); xiao-lu.li@connect.polyu.hk (X.L.); yiu-hong-97.wong@connect.polyu.hk (Y.-H.W.); 2 Photonics Research Institute, The Hong Kong Polytechnic University, Hung Hom, Kowloon, Hong Kong SAR, China

**Keywords:** wearable technologies, wearable music instrument, pressure sensors, motion capture, music learning, musicians, gyroscope, accelerometer

## Abstract

This paper explores the innovative concept of using wearable technologies as a medium for musical expression. Special emphasis is placed on a unique wearable device equipped with motion, touch, and acceleration sensors, which can be used as a wrist strap, hand strap, or surface drum pad. The aim is to create a new musical instrument that simplifies music learning and expression and makes them more intuitive. The wearable device contains 32 individual touch-sensitive pressure sensors, a nine-axis inertial-measurement-unit motion sensor, and various light-emitting diode and vibrational haptic-feedback components. The inclusion of tactile and intuitive features in the wearable device enhances the musical experience of users by enabling engaging interaction. Consequently, it is believed that this groundbreaking technology has significant potential to contribute to the field of music, providing musicians with a versatile and intuitive instrument that facilitates their creative expression.

## 1. Introduction

People have been making music throughout recorded history, using all kinds of instruments, from ancient flutes in 80,000 BC to Babylonian bells in 2000 BC [1]. Even today, people can still make music with nothing but their own bodies, as seen in body percussion. This idea—that anyone can make music—has driven the recent development of wearable technology designed for music creation.

Traditional ways of teaching music, which focus mostly on classical instruments, are changing. While these instruments have much to offer in terms of sound and skill, they can be difficult and expensive to learn. Their complexity can discourage those who want to learn music, especially considering issues like cost and lack of easy access to lessons. Recent data show that as many as 31% of adults and children decide not to learn to play classical instruments, a statistic that points to problems in the current approach to music education [2]. On the other hand, the burgeoning market in electronic musical instruments, which includes technologies such as finger drum pads and virtual music software, reflects the growing appetite for more accessible means of musical expression. This market was valued at US $678.9 million in 2022 and is projected to grow by another 50% within the next decade. The shift towards electronic instruments is part of an emerging trend towards simplicity and immediacy in music creation [3]. The growing convergence of wearable technology and music-making reflects a demand for simpler and more accessible means of musical expression. With the worth of wearable tech industry anticipated to reach an estimated US $931.31 billion by 2030, a new horizon of transformative possibilities lies before us [4]. Wearable technology presents an opportunity to bridge the accessibility gap in musical expression by providing a new way for anyone to make music. 

Music creation through movement is not a new concept, and it has been reimagined through recent developments in wearable music technology [5]. Researchers have explored various methods for using gesture data, sensory data, and multisensory data from instrumental performers to create personalized music materials [6,7]. For example, Marlon et al. investigated physical gestures from the perspective of computer-aided composition (CAC) and proposed solutions and implementation for the integration of gesture signals as musical materials [8]. Additionally, Gunther et al. built a compositional language using the sense of touch and created a vibrotactile composition suit implementing that tactile composition system [9]. Furthermore, new musical instruments with gestural sensors have also been introduced [10], and electronic variants have transformed the musical gestalt of player–instrument and listener–music relations [11]. These advancements showcase the potential of utilizing gesture and sensory data for musical expression. Such approaches can enhance the interaction between instruments and instrument players. As a result, they have the potential to expedite the development of personalized music materials.

However, the development of wearable music instruments poses significant challenges due to various requirements, including miniaturization, seamless integration of sensors, efficient signal processing, low latency, reliable connectivity, effective power management, optimal user experience, and affordability. The implementation of this technology requires the design of a wearable device equipped with all these features, which necessitates expertise in electronics, sensor technology, signal processing techniques, and human-computer interaction. Additionally, considerations such as optimization of power consumption, intuitive interface design, and cost-effectiveness further contribute to the complexity involved in creating a successful wearable music instrument. Therefore, the development process typically involves iterative prototyping, rigorous testing, and continuous refinement to create a device that successfully addresses these multifaceted challenges.

Pioneering commercial devices such as DrumPants, Freedrum, Mi.Mu Gloves, and Remidi gloves have graced the global market since 2008 and have been primarily backed by community-funding platforms such as Kickstarter and Indiegogo. These inventions emphasize the employment of motion sensors for music creation and draw in early adopters, a group consisting of enthusiastic learners with different musical backgrounds and knowledge levels [12]. The integration of touch-sensitive pressure sensors is a secondary innovation in these devices [13] that allows users to augment their musical expression through finger and palm-pressure controls, as well as through body movement. However, these sensors have serval limitations. For instance, DrumPants digitalizes the human body by replicating the typical movements of someone drumming on a drum kit [14]. However, it experiences latency issues when large numbers of tracks and effects are added. Additionally, although Freedrum offers valuable insight into the mechanics of virtual music creation because it utilizes a gyroscope and accelerometer, it should be noted that the sensors are attached to the drumsticks rather than directly to the body of the wearer. The Mi.Mu and Remidi e-gloves manifest the essence of wearable technology for music creation by integrating flex and pressure sensors, as well as nine-axis inertial measurement units (IMUs) [15], but they are expensive.

This project aims to address the limitations of the currently available wearable music devices by introducing an innovative and more affordable miniaturized wearable music instrument that makes music learning more accessible and enhances the user experience. It can be worn as a wrist strap, hand strap, or surface drum pad, offering enhanced versatility compared to traditional glove-style devices. The primary objective, to provide musicians and enthusiasts with a unique and intuitive method of interacting with music, is achieved through the simplification of traditional musical instruments and feedback systems. This approach is believed to enhance both musical expression and creativity. The device incorporates advanced sensor technology, including 32 touch-sensitive pressure sensors and a nine-axis inertial measurement unit motion sensor into the wearable device, enabling precise motion and touch detection. Additionally, the wearable device features a light-emitting diode and vibrational haptic feedback components, providing tactile and intuitive feedback. Through the integration of these components, the wearable device offers an immersive and interactive musical experience. Furthermore, this project also seeks to explore the potential of wearable technology to drive musical innovation. By creating a symbiotic relationship between musicians and their music through this novel instrument, the project aspires to revolutionize the music industry and empower users to realize their full artistic potential.

## 2. Materials and Methods

This study employs a practice-based-design research methodology, with a specific focus on qualitative analysis. The research process began with an in-depth examination of related products and technologies through case studies, aiming to identify their strengths and limitations. Six case studies were included in this research: (1) KaiKU gloves, (2) Mi.Mu gloves, (3) Remidi T8 gloves, (4) DrumPants, (5) AUUG wearable device, and (6) Mogees. Following the case-study analysis, two innovative prototypes were designed and developed. The design of these prototypes, which are intended to be worn on the hands, were informed by the insights gained from the case studies. The design process was iterative, with each version of the prototypes being refined based on feedback and testing.

Subsequently, wear trials of the prototypes were conducted. These trials were designed to gather data for the evaluation and analysis of the design. A validation process was employed in which subjects were recruited to try the prototypes and evaluate their performance in playing music. Specifically, subjects were asked to play a prechosen song, and each subject’s ability to accurately reproduce the melody, rhythm, and pitch was assessed. Additionally, measurements of hand size and dexterity were taken during these trials. The objective was to understand the variations in these factors and how they could influence the design of a new wearable device that could fit all hands universally and enhance interactivity. The data collected from these wear trials were then analyzed to assess the effectiveness of the prototypes and to inform further design improvements. This iterative process of design, testing, and refinement is central to the methodology of practice-based-design research employed in this study.

## 3. Results and Discussion

### 3.1. Case Study

#### 3.1.1. KAiKU Gloves

Developers have been experimenting with the expression of music using not only software, but also hardware, for many years. One of the more recent inventions is a handheld device that translates not only motion, but also pressure from the fingers [16]. The device works impressively well. Naturally, this product was not made for the masses. Instead, like the Mi.Mu gloves described below, it was made for professionals. Its use is complex, requires specialized training, and cannot be made user-friendly, and that is why it was never widely adopted.

#### 3.1.2. Mi.Mu Gloves

Among similar research and commercial products, the Mi.Mu gloves were among the first to use flex sensors in music production [15]. They are also equipped with pressure sensors and a shared nine-axis inertial measurement unit. The combination of these technologies in the gloves allows not only for the creation or triggering of sound, but also for the production of coherent and intelligible songs. Moreover, the gloves have a state-of-the-art design and a highly responsive electronic schematic that makes use of all five fingers and all axes of the gyroscope to detect acceleration. In a prime demonstration of the possibilities of wearable music technology, music artist Chagall showcased how using individual finger flex sensors, paired with the multidirectional controls of the Mi.Mu gloves, can create a bassline loop, percussive drumbeat sounds, and even vocal sound effects. Over a span of 3 min and 15 s in her video, Chagall provides a compelling demonstration of the inherent motion of music and illustrates how—depending on the predefined parameters of the New Interface for Musical Expression (NIME)—a beginner can swiftly transition into performing as a virtual musician [15]. While the product garnered significant attention, its primary drawback lies in its pricing. Considering that a single Mi.Mu glove costs at least US $1500, it is hardly affordable to the general public, except to dedicated electronic-music or wearable-technology enthusiasts. In an age in which we aspire to make new wearable technologies for music accessible, this pricing is a deficiency of the Mi.Mu gloves.

#### 3.1.3. Remidi T8 Gloves

Similarly, the Remidi T8 gloves were developed by integrating eight pressure sensors and a MIDI wrist controller into ultra-light spandex gloves. Additionally, when these gloves are synchronized with haptic-feedback technology, musicians are able to play a virtual keyboard in the air by moving their fingers and hands [17]. Simultaneously, the gloves provide tactile feedback to simulate the sensation of pressing physical keys. This functionality enables the gloves to function as an independent instrument that mimics traditional musical instruments. It is believed that this wearable instrument offers a compact and immersive method for playing classical piano pieces, facilitating convenient practice sessions and live performances. However, it is important to note that the tight fit of these synthetic gloves may compromise their breathability, which could potentially result in the accumulation of perspiration within the glove and thus in malodor and discomfort.

#### 3.1.4. DrumPants

DrumPants is a vest with integrated velocity-sensitive pressure sensors and a digital interface that is designed to replicate playing the drums, but on the body [14]. Additionally, the sensors can be affixed to users’ chosen body parts with elastic straps and Velcro. Upon touch, each sensor triggers a designated MIDI output associated with a specific sound or effect. Thus, users can press on the surface of the vest to change pitches, bow virtually, and trigger various string articulations. For instance, striking one’s lap simulates hitting a snare drum, and stamping one’s foot recreates hitting a bass drum. Even with these simple elements alone, a user can mimic playing a drum kit, as aptly demonstrated in the Kickstarter video for DrumPants. It enables drummers to practice virtually and experiment with electronic sound effects, thus expanding their creative horizons. These features serve as a benchmark for the proposed wearable music instrument.

#### 3.1.5. AUUG Wearable Device

The AUUG is a wearable device for the hand that combines a wristband and holder for a smartphone [18]. Musicians can strum, pluck, and bend virtual strings to produce realistic guitar sounds. The sensors of the wristband detect finger movement and pressure, which allow for intricate playing techniques. “WristStrum” is a portable and expressive alternative to traditional guitars that enables musicians to create music outside traditional settings. However, it is not an independent instrument in its own right, but is attached to traditional instruments to enrich musical expression.

#### 3.1.6. Mogees

Mogees, an app and vibration sensor, is a splendid example of how a MIDI device in its simplest form can make an enormous impact on the community [19]. For several years, it was common for users to attach their Mogees to a large number of different materials and objects and create intelligible electronic musical sounds by transforming and translating the natural frequencies of the materials and objects. Mogees offers users the ability to generate creative, playable music, but without the regularity and organization associated with musical instruments.

### 3.2. Design and Development of Prototypes

Based on the case studies, two novel prototypes were designed and developed to create music through hand motions. The concept design was arguably influenced by, but not directly copied from, the designs of the Mi.Mu and Remidi gloves. Additionally, the placement of all sensors in the hand was completely novel, introducing a unique configuration. The details are as follows.

#### 3.2.1. Prototype 1: Strap with Four Pressure Sensors

**Figure 1:**
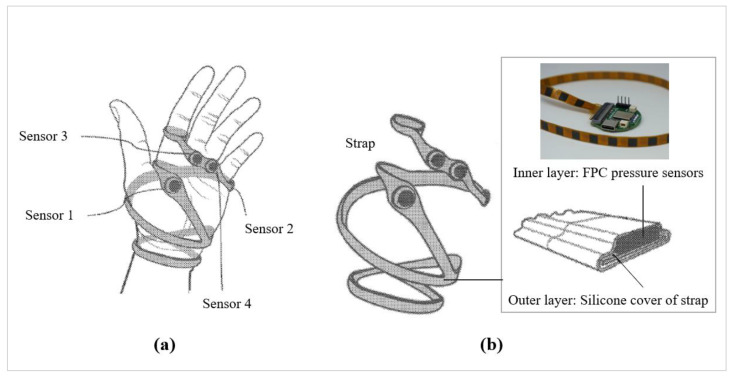
Design details of Prototype 1: (**a**) locations of pressure sensors and (**b**) cross-section of the strap.

The aim in creating the prototype was to optimize for interactivity. Therefore, the first prototype was designed to function as both a surface controller and a piano, enabling users to interact with it by pressing on its surface. However, this implementation proved unsuccessful, leading to the development of a second prototype to address its limitations. As shown in Figure 1, the design incorporated a silicone strap that wrapped around the palm twice and split around the thumb. A silicone cover was chosen for its ability to facilitate stable signal transmission and its ease of cleaning [20]. To fulfill the criteria of minimal space utilization and high sensitivity to pressure, four individual piezo pressure sensors (FM0008-01 Long FPC-FSR) from various Alibaba suppliers (China) were used as the flexible printed circuit (FPC) pressure sensors. The sensors were positioned at specific locations: (1) on the palm, (2) on the side of the palm, and (3) on the metacarpal of the middle finger and (4) on the ring finger. Additionally, the strap was securely fastened around the index finger using a loop design.

**Figure 2:**
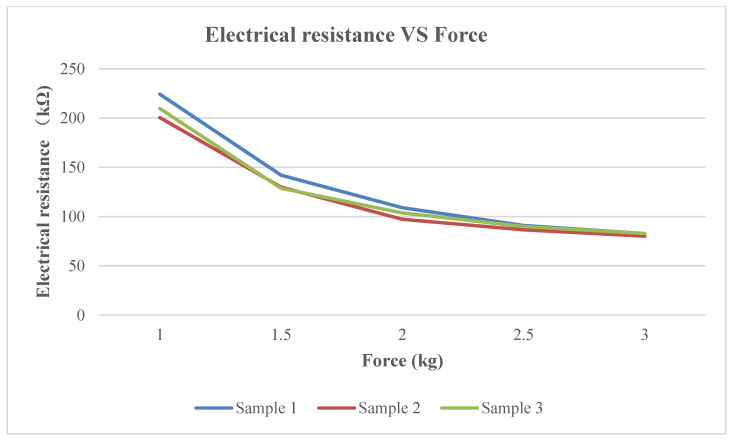
Comparison of electrical resistance in different pressure-sensor samples.

Three different FM 0008-01 Long FPC-FSR pressure-sensor samples with varying resistance levels were tested at different intervals using varying amounts of force (kg). Sample 1 featured a thicker top layer of filament, while Samples 2 and 3 had similar thicknesses but were made with slightly different filament materials. As shown in Figure 2, Sample 1 displayed a greater range of resistance values, from 1 kg to 3.5 kg, indicating higher sensitivity and increased responsiveness to user touch. These results indicated that Sample 1 could be triggered at the lowest weight, which was only 1 kg.

Despite its effectiveness in distinguishing between hand motions such as a slap, a chest-slap, and a clap, Prototype 1 encountered limitations in its functionality as a wearable hand controller and piano-like device. For example, it was necessary to affix a significant number of pressure sensors to the strap. These manufacturing challenges ultimately prompted the development of the second prototype.

#### 3.2.2. Prototype 2: Strap with Full Length of Touch-Sensitive Pressure Sensors

**Figure 3:**
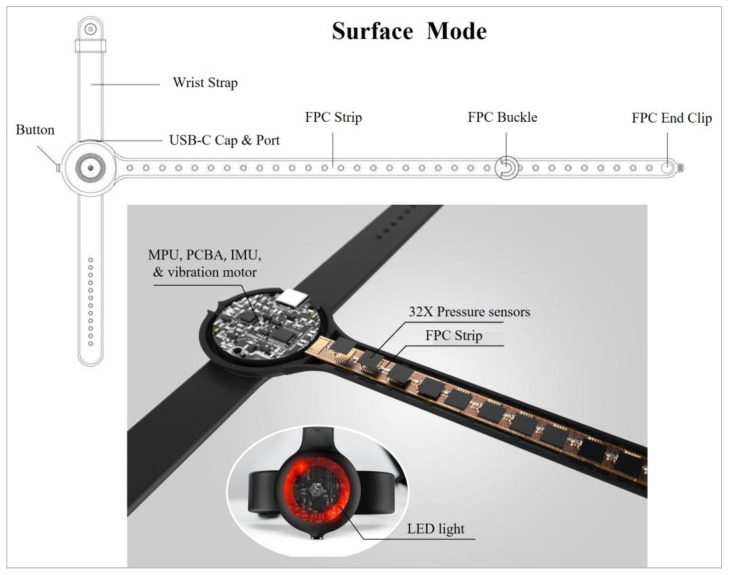
Design details of Prototype 2.

The second prototype was constructed based on the same design concept as Prototype 1 and was wrapped around the hand in a similar manner. However, unlike Prototype 1, which has only four FPC pressure sensors, the second prototype contained 32 individual touch-sensitive pressure sensors along the strap (Figure 3). The incorporation of a greater number of pressure sensors expands the range of sound and light effects, as in a piano wherein each note carries a distinct sound. This modification enables Prototype 2 to function as a surface piano or drum pad while still being a wearable device that covers both the palm and knuckles of the hand for dual-control access by the fingers and the back of the hand. It is believed that this design offers enhanced versatility compared to traditional glove-style devices. Additionally, special attention was given to addressing breathability and hygiene problems, ensuring user comfort by preventing sweat accumulation and the associated clingy feeling. Furthermore, the motion processing unit (MPU) and printed circuit board assembly (PCBA) on the motherboard were used to define the origin point of the nine-axis motion controller. These components detect hand movements and translate them into musical parameters such as pitch or volume. Moreover, the inertial motion unit (IMU) measures the gyroscopic, acceleration, and magnetometer values of the user’s arm gestures, enabling dynamic control over musical elements [21]. To improve the user experience and increase the degree of interactivity that is possible, Prototype 2 is also equipped with a 12-diode chip on board (CoB) LED. With this LED, the device displays different lights for each gesture when users rotate their hands between two different gestures. Meanwhile, the vibration motor provides tactile feedback by vibrating when the user squeezes a pressure sensor. These additions allowed for the exploration of motion touch and haptic feedback, utilizing lights and vibrations to enrich the process of musical creation. 

The integration of motion, touch, and acceleration sensors in the musical interface enriches the user experience with enhanced expression, control, and responsiveness. For example, the motion sensors enable users to physically express themselves and create music through gestures and motions, adding a tangible and embodied dimension to the experience. The touch sensors facilitate precise interaction, allowing users to shape the musical output according to their intentions. Additionally, the dynamic control provided by the acceleration sensors empowers users to infuse their music with expressive gestures, imbuing it with a sense of liveliness and responsiveness.

**Figure 4:**
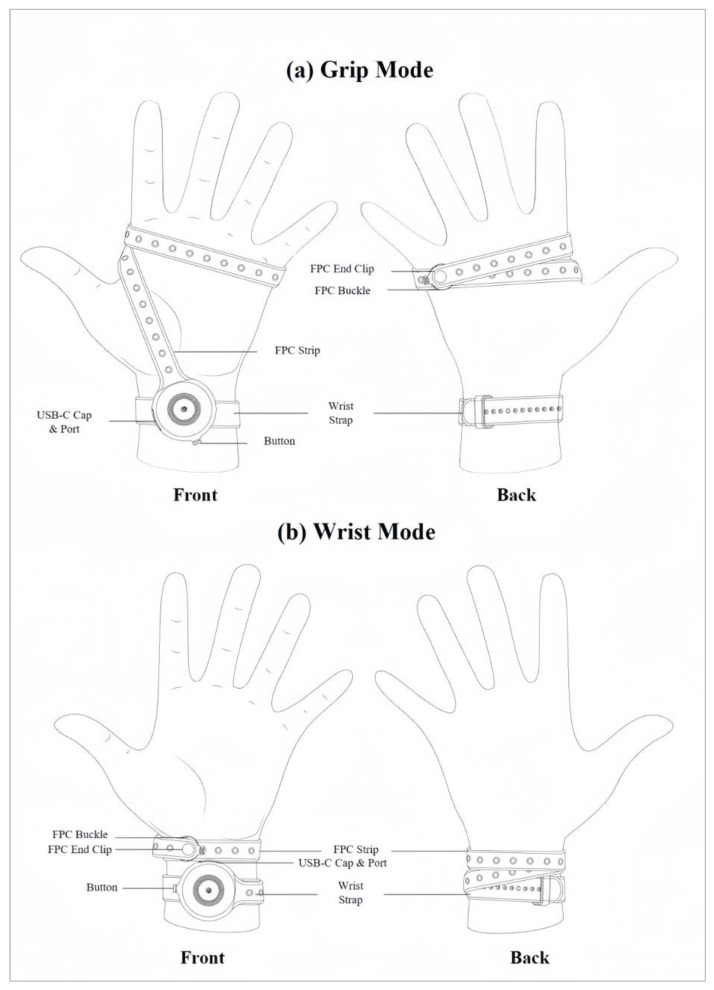
(**a**) Grip and (**b**) wrist modes of Prototype 2.

In the grip mode, the strap covers the palm, the side of the palm, and all the metacarpals of the hand (Figure 4a). In the wrist mode, the device can be worn as a bracelet that does not need to be removed completely when it is not in use (Figure 4b). In both cases, the device enables music creation, with the main distinction being that in wrist mode, the fingers of the same hand cannot be utilized to trigger the pressure sensors, as those sensors are positioned on the wrist.

The device employs a motion sensor to detect motion across all wearing modes. However, the range of motion varies depending on the specific mode chosen. In both the wrist and grip modes, the device is capable of capturing the full range of motion of the wrist, allowing users to leverage their natural hand and wrist movements for seamless control and interaction. Conversely, in surface mode, the motion control of the device is constrained, and the device primarily recognizes motion when the user deliberately lifts the device from the surface and adjusts it. In this mode, the focus shifts towards intentional manipulation and repositioning of the device, rather than continuous motion capture. These distinctions in motion sensing in different wearing modes empower users to adapt their interaction with the device, tailoring it to their preferred mode and intended use. This information has been incorporated into the manuscript, providing further clarity regarding the device’s capabilities with regard to motion sensing.

### 3.3. Making Music through Movement

**Figure 5:**
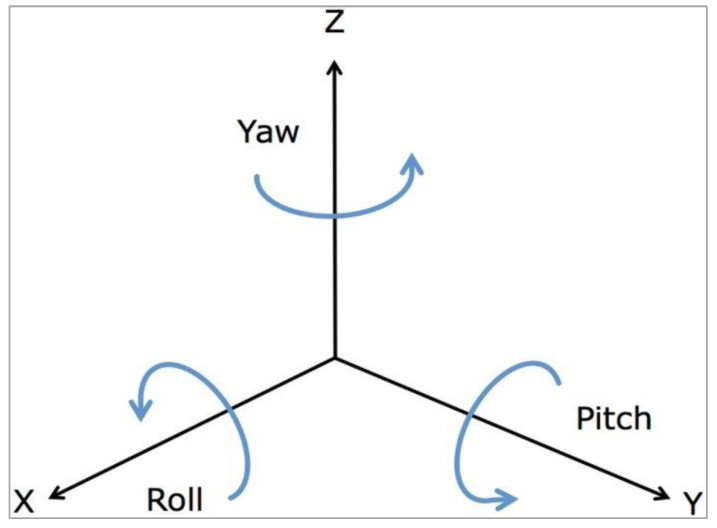
Making music using movement.

There are infinite ways in which motion can translate into music. One method involves subdividing the angular data from the gyroscope and mapping each angular range to a specific set of musical outputs (MIDI outputs). One arrangement that employs this method maps 90° angles onto block triad chords of major and minor scales (Figure 5). In this study, this concept is referred to as Motion Static. Each 90° angle corresponds to a specific gesture, and the corresponding gestures are shown in Table 1 [22]. Moreover, within the range of each 90° gesture, the device triggers a different MIDI output. In this case, the outputs are defined as the block chords of the major and minor scales of Western music. The experiments were conducted for the left hand only.

In addition to the predefined values for 90° angles, an acceleration threshold in the negative Z direction is set so that the user can switch between block (all notes played together) and arpeggiated (all notes played separately in a sequence) chords. A third and final condition was implemented using pressure sensors. If the pressure threshold is exceeded, all sound outputs are muted.

### 3.4. Mobile Software Design

**Figure 6:**
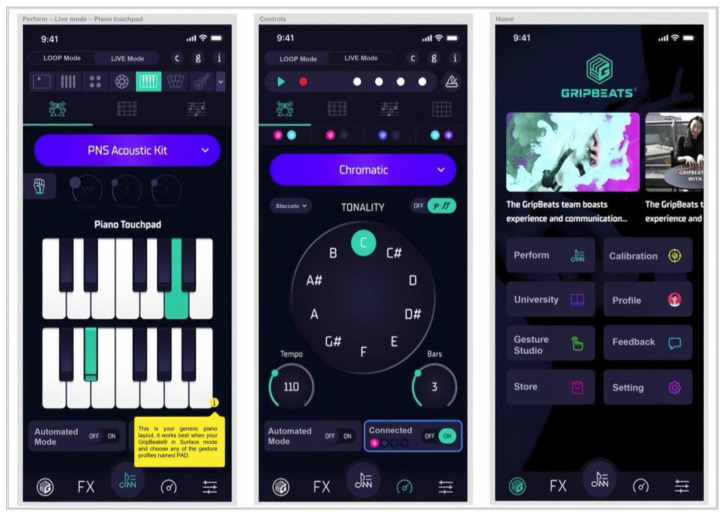
Interface of the mobile software design with instructions.

An essential component of a wearable music controller involves providing users with an interface and instructions on how to effectively utilize the controller. Consequently, it is imperative to design a tailored mobile application (app) to fulfill this purpose. The app consists of three distinct pages: a piano page for visualizing the music played, a turn-dial page for editing the types of sounds and their effects, and a home page for organization. This app is user-friendly enough so that novices can immediately start to produce music while gaining an understanding of the theoretical aspects (Figure 6).

**Figure 7:**
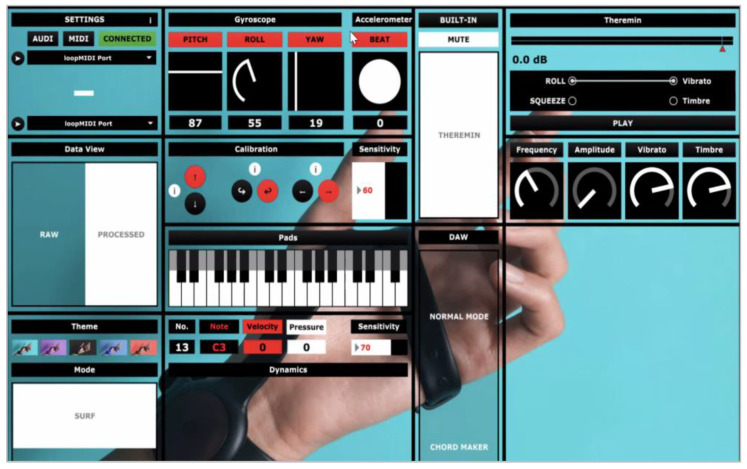
Modified interface.

The app was further enhanced after a trial run with users, who indicated that the ability to edit the actual output parameters of their devices would be ideal. Thus, a desktop application that allows users to do so was subsequently created for Windows 10/11 and Mac. Controls to map the individual gyroscopic values for pitch, roll, and yaw, along with the defining values for the minimum and maximum range positions for each movement, are shown in Figure 7. Feedback was received from over 20 users, and enhancement of the preexisting Desktop App continued with the addition of more features and functions of control. For example, a “Theremin” was implemented; this feature essentially functioned as an in-app synthesizer that allowed users to create sounds directly from the app without the need to connect it to third-party Digital Audio Workstation (DAW) software. Users tended to be between the ages of 18 and 42 and included mostly tech-savvy musicians, but the group also included complete music beginners interested in making music for the first time, especially through movement.

### 3.5. Design Evaluation

**Figure 8:**
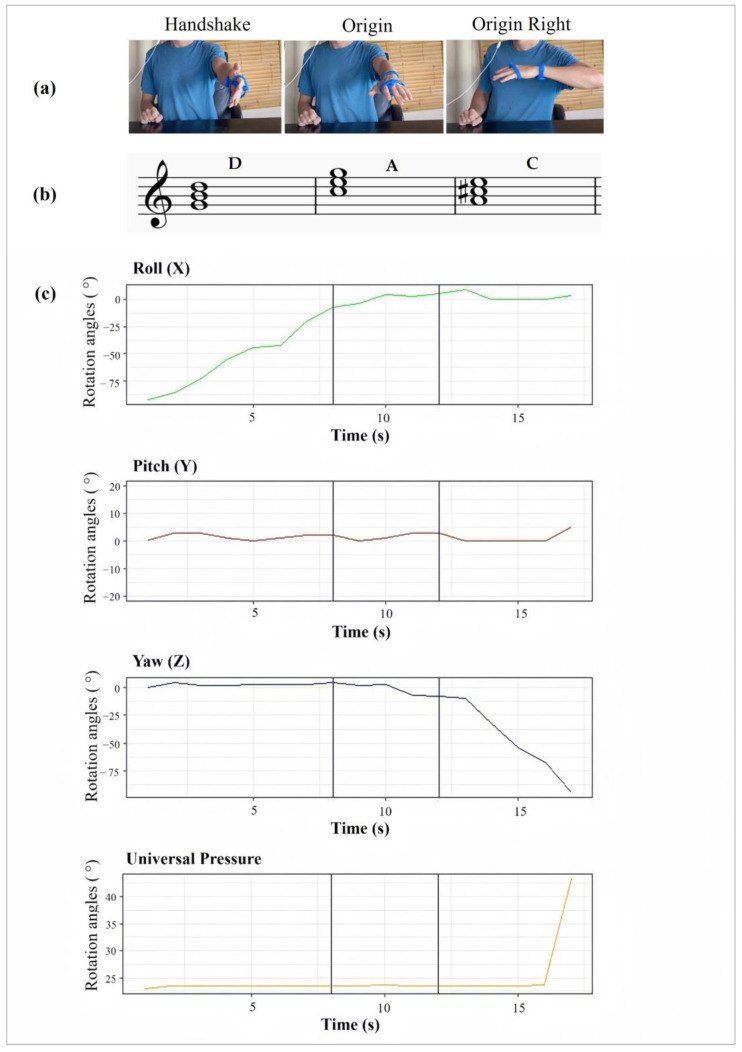
(**a**) Gestures mapped to (**b**) major chords and (**c**) rotation angles.

A wear trial was implemented to evaluate the design of the prototypes. In this trial, to evaluate the prototypes, different arpeggiated chords were played according to three different gestures: handshake, origin, and origin right (Figure 8). The wear trial was conducted with over a dozen members of the public from the university who were selected through various competitions and through word of mouth, including, for example, friends of friends. Most users were interested in the product itself, which is why it was possible to conduct the trials using volunteers.

In this example, the flow of the gestures from Handshake to Origin, and then to Origin Right, was recreated to play a block chordal progression with GarageBand, a Digital Audio Workstation (DAW) developed by Apple. Throughout the performance, the changing signal data from the nine-axis IMU sensor (X, Y, and Z rotation angles) and pressure sensor are wirelessly transmitted to the computer via gestures.

However, latency was a challenge at some points, as it affected the connection between gestures and music. Additionally, the Motion Static profile did not include a large number of motions, i.e., users were meant to arrange their hands only in specific positions and not to perform any ongoing or continuous motion. As a result, practically all 90° angles output the designated musical output. Gyroscopic accuracy was more of an issue than latency, as the three-axis values showed particular sensitivity. Therefore, a threshold was implemented to limit accidental triggering of the X, Y, and Z values during slow and steady 90° gestures. This setting is also known internally as an offset calibration.

## 4. Challenges of Developing Wearable Music Technology

The development of wearable music technology is not predominantly hindered by the manufacturing of the hardware involved [23,24,25]. Many startups worldwide have developed an assortment of hardware kits that focus on electronic music controls. Although many of these startups grapple with producing commercially viable hardware, production is not the core predicament. Similarly, the creation of software that converts sensor data from the hardware into sound and music is typically uncomplicated. For instance, most software engineers have the capability to utilize basic MIDI encoding that enables a user to generate prerecorded samples or start a sine wave [26,27].

However, the real challenges in developing wearable music technology emerge when considering the creation of an intelligible musical instrument that can facilitate the production of coherent songs. These songs should uniquely amalgamate distinct musical rhythms, melodies, sounds, and effects. A wearable technology device that simply generates a sound based on a singular gesture does not serve as a universally comprehensible musical instrument. It is this criterion that differentiates a musical toy from a bona fide musical instrument.

Creating a device that transforms specific movements or gestures into unique musical sounds may initially seem exciting. However, it is crucial to consider how well users comprehend these actions and the music they generate [28]. One potential solution lies in personalization, wherein users have the ability to define their output parameters based on pre-provided input-gesture values. Without flexibility that allows users to gain personal understanding through trial-and-error mapping of gestures to musical outputs, the art of creating music through motion and touch can become simply another repetition of existing technology, rather than an intuitive, personalized learning experience. This concept of personalization has been explored in the prototypes developed in this study, with the aim of innovating in the field of wearable music technology [29].

## 5. Limitations of Experiments and Future Trends

### 5.1. Limitations of Experiments

While the findings of this study are encouraging, it is important to acknowledge several limitations that were encountered. The first limitation is the time-delay issue encountered during the study. The delay between the musician’s movements and the corresponding musical output could disrupt the rhythm of the music and limit real-time music creation. Future research should aim to minimize this delay to ensure a more fluid musical experience.

The second limitation pertains to the unstable signal transmission experienced by the prototypes developed in this study. This instability, which was likely due to the wireless nature of the devices, could potentially interrupt the process of musical creation. Future research should therefore prioritize enhancing the stability and reliability of signal transmission.

The third limitation involves the suboptimal responsiveness of the pressure sensors. There were instances in which the sensors did not respond as anticipated, potentially hindering the musician’s ability to accurately control the musical output. To address these issues, future iterations of the device could incorporate higher-quality sensors or refine the sensor-calibration process.

These proposed enhancements are intended to provide a smoother musical experience and superior control over the instrument. As we consider future directions, this study underscores the dynamic nature of music technology and the ways in which it can empower individuals. The prototype offers not only a means of making music, but also an immersive learning experience that can benefit both musicians and non-musicians. It challenges traditional limitations and opens up new possibilities for creative expression. The future of music technology will likely continue to push boundaries and explore innovative ways of creating music.

### 5.2. Future Trends

Despite the prevailing opinion among traditionalists that a musical instrument not invented prior to 1950 is considerably primitive in terms of music-making capability, modernists and their latest musical instrument inventions have evolved beyond such conservative notions. The music-technology-products industry alone has fostered more talent in the music community, even within the definitions of the traditionalists themselves, than their classical instruments ever did.

Even if traditional musical instruments are ultimately abandoned due to the time, effort, and cost required to learn how to play them, there is still a good deal of public enthusiasm and interest in making music. Most people believe that the application of this type of wearable technology is limited to music-technology enthusiasts and prospective musicians. However, many individuals, especially young people, are now seeking new means by which to share their creative and expressive talents on social-media platforms like TikTok, which are used by billions of users and influencers each month. Therefore, the question is never “Will they use it?”, but rather “Can they use it?”. The venture accelerators, also known as startup accelerators, are all asking the wrong questions. Non-musicians can indeed become musicians. Moreover, musicians from traditional classical or digital backgrounds can transcend their current limitations and use technology to enhance their abilities.

The prototype in this study is capable of providing such individuals with another outlet through which to do so. It offers an immersive learning experience that schools and educational institutions can use not only to develop musical skills, but also to reap the cognitive benefits of this technology. Additionally, it challenges the traditional limitations of music with its ability to simulate almost all existing means of making music using movement and touch alone. By combining some of the more complex theories and intricacies of musical creativity with the sublime simplicity of hand motion, the prototype was able to engage music enthusiasts of all backgrounds and beliefs. Aside from making music, the prototype may have other functional uses, such as empowering those who have both physical and mentally challenges, for example, individuals with ADHD. The ability to create music through movement means that those who face such limitations will be able to stimulate their creative musical abilities and express themselves if they are not able to do so vocally or physically. It is believed that the future of music technology is likely to continue pushing the boundaries imposed by traditional instruments and to explore new ways of creating music.

## 6. Conclusions

In conclusion, this study has effectively pioneered the application of wearable technologies as a novel medium for musical expression. The two prototypes, both equipped with pressure sensors and designed to be worn on the hand, demonstrated the potential to revolutionize music creation and experience. The first prototype served as an initial demonstration of a wearable surface controller and piano, showcasing the potential of wearable technology to create a highly interactive and intuitive musical instrument. The second prototype, with its increased number of pressure sensors and additional features such as a motion processing unit, LED lights, and a vibration motor, further enhanced the musical experience by providing a more versatile and engaging interface. These groundbreaking devices have the potential to significantly contribute to the field of music, offering musicians a new, intuitive, and versatile instrument for creative expression. Future research will involve exploring the potential of wearable technology to expand individuals’ musical capabilities and transcend traditional limitations. Potential applications include leveraging wearable devices to provide immersive learning experiences in schools and educational institutions, enabling individuals with diverse backgrounds and abilities to engage in music creation. Additionally, wearable technology holds promise for empowering individuals with physical and mental challenges to stimulate their creative musical abilities and engage in self-expression. This ongoing trend in music technology aims to push the boundaries imposed by traditional instruments and explore new avenues for creating music.

## Figures and Tables

**Table 1:** Motion Static settings and MIDI Note Output.

**Gesture** **(Point of View:** **Reviewer)**	**Orientation** **of X, Y, Z (°) & MIDI Note Output**	**Gesture** **(Point of View:** **Reviewer)**	**Orientation** **of X, Y, Z** ** (°) &** **MIDI Note Output**
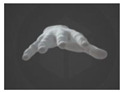 Origin	0, 0, 0 C, E, G	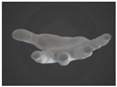 Reverse Origin	−180, 0, 0 A, C, E
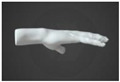 Left	0, 0, 90 Db, F, Ab	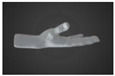 Reverse Left	−180, 0, 90 Bb, Db, F
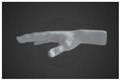 Right	0, 0, −90 A, Db, E	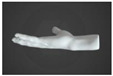 Reverse Right	−180, 0, −90 Gb, A, Db
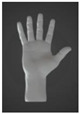 Up	0, 90, 0 A, Db, E	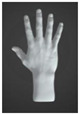 Reverse Up	−180, 90, 0 Gb, A, Db
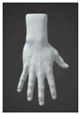 Down	0, −90, 0 Eb, G, Bb	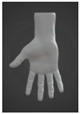 Reverse Down	−180, −90, 0 C, Eb, G
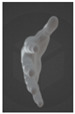 Handshake	−90, 0, 0 G, B, D	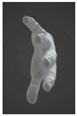 Reverse Handshake	90, 0, 0 E, G, B
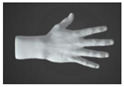 Handshake Left	−90, 0, 90 Gb, Bb, Db	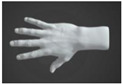 Reverse Handshake Left	90, 0, 90 Eb, Gb, Bb
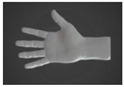 Handshake Right	−90, 0, −90 D, Gb, A	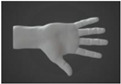 Reverse Handshake Right	90, 0, −90 B, D, Gb
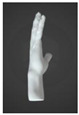 Handshake Up	−90, 90, 0 E, Ab, B	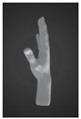 Reverse Handshake Up	90, 90, 0 Db, E, Ab
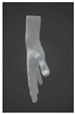 Handshake Down	−90, −90, 0 Ab, C, Eb	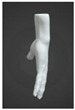 Reverse Handshake Down	90, −90, 0 F, Ab, C

## Data Availability

The datasets that were generated and/or analyzed during the current study are available upon reasonable request from the corresponding author.
